# Synergistic Effects of Temperature and Cooling Rate on Lamellar Microstructure Evolution and Mechanical Performance in Ti-44.9Al-4.1Nb-1.0Mo-0.1B-0.05Y-0.05Si Alloy

**DOI:** 10.3390/ma18194641

**Published:** 2025-10-09

**Authors:** Fengliang Tan, Yantao Li, Jinbiao Cui, Ning Liu, Kashif Naseem, Zhichao Zhu, Shiwei Tian

**Affiliations:** 1 School of Materials and Environmental Engineering, Hunan University of Humanities Science and Technology, Loudi 417000, China; hnrw3113@163.com (F.T.);; 2 Technical Center of Hunan Valin Lianyuan Iron and Steel Co., Ltd., Loudi 417009, China; 3 Institute of Engineering Technology, University of Science and Technology Beijing, Beijing 100083, China

**Keywords:** TiAl alloy, heat treatment, cooling rate, high-temperature tensile, γ allotriomorphs

## Abstract

TiAl alloys are ideal candidates to replace nickel-based superalloys in aero-engines due to their low density and high specific strength, yet their industrial application is hindered by narrow heat treatment windows and unbalanced mechanical performance. To address this, this study investigates the microstructure and mechanical properties of Ti-44.9Al-4.1Nb-1.0Mo-0.1B-0.05Y-0.05Si (TNM-derived) alloys hot-rolled in the (α_2_ + γ) two-phase region. The research employs varying heat treatment temperatures (1150–1280 °C) and cooling rates (0.1–2.5 °C/s), combined with XRD, SEM, EBSD characterization, and 800 °C high-temperature tensile tests. Key findings: Discontinuous dynamic recrystallization (DDRX) of γ grains is the primary mechanism refining lamellar colonies during deformation. Higher heat treatment temperatures reduce γ/β phases (which constrain colony growth), increasing the volume fraction of lamellar colonies but exerting minimal impact on interlamellar spacing. Faster cooling shifts γ lamella nucleation from confined to grain boundaries to multi-sites (grain boundaries, γ lamella peripheries, α grains) and changes grain boundaries from jagged and interlocking to smooth and straight, which boosts nucleation sites and refines interlamellar spacing. Fine lamellar colonies and narrow interlamellar spacing enhance tensile strength, while eliminating brittle βo phases and promoting interlocking boundaries with uniform equiaxed γ grains improve plasticity.

## 1. Introduction

TiAl alloys, with their low density, high specific strength, and excellent high-temperature oxidation resistance, are considered promising candidates to replace nickel-based superalloys in aerospace engine hot-end components (e.g., turbine blades) [1,2]. In recent years, the forged Ti-43.5Al-4Nb-1Mo-0.1B alloy has been successfully applied in the turbine blades of Boeing 787 engines [3], significantly reducing blade weight while improving engine fuel efficiency. However, large-scale industrial adoption of these alloys remains hindered by two major challenges: (1) a narrow hot working and heat treatment process window, as their microstructure is extremely sensitive to temperature, holding time, and cooling rate; and (2) the need to further enhance comprehensive high-temperature service performance (e.g., creep resistance, fracture toughness). Given the strong correlation between microstructural morphology (e.g., lamellar colony size, interlamellar spacing, and grain boundary characteristics) and high-temperature mechanical behavior, extensive research has been conducted globally to optimize the microstructural features of TiAl alloys [4,5]. Addressing the dual objectives of achieving precise control over lamellar structures via simple and efficient heat treatment processes and further improving service performance remains both a critical scientific challenge and a key breakthrough for advancing industrial applications of TiAl alloys.

From the perspective of heat treatment process optimization, Bernal et al. [6] observed that increasing the heat treatment temperature of TiAl alloy shortens the dissolution time of the γ phase. Above 1260 °C, spherical γ phase precipitates preferentially at lamellar colony boundaries, enhancing ductility. Huang [7] reported that elevated temperatures promote grain coarsening, whereas air cooling (compared to furnace cooling) yields more uniform and refined lamellar structures. Quan [8] achieved a fully γ/α_2_ lamellar structure via a two-step heat treatment and observed a pearlitic-like structure when aging above 800 °C. Qiang [9] designed a three-step process for high-Nb TiAl alloys: short-term holding in the β phase region to eliminate segregation, annealing in the α + β region to homogenize the microstructure, and low-temperature aging to precipitate γ lamellae, achieving concurrent improvements in strength and toughness. Tian [10] employed cyclic heat treatment to refine the average lamellar colony size of TiAl sheets and eliminate β/B_2_ phases. These studies collectively confirm that heat treatment temperature and cooling rate are pivotal for controlling microstructural features (e.g., lamellar colony size, phase composition) in TiAl alloys. However, most existing works rely on complex multi-step processes [8,9] or cyclic treatment [10], which are less suitable for industrial mass production due to high cost and long cycle. Furthermore, few studies focus on tailored TiAl alloys doped with trace elements (e.g., Y, Si), and the intrinsic mechanisms (e.g., γ lamella nucleation sequence, lamellar fragmentation driver) underlying microstructural regulation remain insufficiently clarified.

In γ/α_2_ lamellar colony structures, smaller colony sizes correlate with a higher density of colony boundaries per unit volume. These boundaries are susceptible to grain boundary sliding (GBS) activation during long-term service, which significantly degrades creep performance [11,12]. With respect to interlamellar spacing effects, reducing the γ/α_2_ interlamellar spacing substantially increases the density of γ/γ and γ/α_2_ interfaces. Such high-density interfaces effectively enhance high-temperature mechanical properties by impeding dislocation motion [12]. Furthermore, narrower interlamellar spacing suppresses fatigue crack nucleation and propagation. Neogi et al. [13] demonstrated through calculations that nanoscale interlamellar spacing optimizes stress distribution at crack tips, thereby substantially improving fracture toughness and crack growth resistance. Nevertheless, existing research primarily focuses on the “microstructure-performance” correlation, while the linkage between heat treatment parameters, core microstructural evolution mechanisms (e.g., discontinuous dynamic recrystallization, DDRX), and lamellar structure control remains unclear.

To achieve effective control of lamellar colony structures in TiAl alloys—especially for the tailored as-rolled TiAl alloy doped with trace Y/Si (distinct from conventional high-Nb TiAl alloys or TNM-derived alloys)—this study proposes a targeted single-step heat treatment process. Compared to the multi-step or cyclic heat treatment reported in previous works, this process not only enables precise manipulation of holding temperature and cooling rate to directionally optimize lamellar colony size, interlamellar spacing, and grain boundary morphology but also clarifies two core original mechanisms: (1) the three-stage nucleation behavior of γ lamellae (from grain boundaries → colony peripheries → α grain interiors); and (2) the discontinuous dynamic recrystallization (DDRX)-dominated lamellar fragmentation law. These findings not only provide critical theoretical insights for tailoring microstructural features to enhance high-temperature mechanical performance but also lay a foundation for optimizing the strength-ductility balance of TiAl alloys, directly addressing the high-temperature service requirements of aero-engine turbine components.

## 2. Experimental Methods

The TNM-derived alloy ingot (Ti-44.9Al-4.1Nb-1.0Mo-0.1B-0.05Y-0.05Si, at%) with dimensions of φ110 mm × 80 mm was remelted three times in a vacuum melting furnace to ensure chemical homogeneity. The ingot was produced using raw materials including Ti-75%Nb alloy, Ti-50%Mo alloy, TiB_2_, high-purity titanium (99.99%), high-purity aluminum (99.99%), high-purity Y_2_O_3_ (99.99%), and high-purity silicon (99.99%). Subsequently, the ingot was held in a resistance furnace at 950 °C for 36 h to achieve microstructural homogeneity.

A TiAl plate with dimensions of 100 mm × 60 mm × 12 mm was cut from the ingot using wire cutting. The TiAl alloy plate was placed in a box-type furnace at 1250 °C and held for 30 min prior to rolling to ensure the deformation temperature remained within the (α + γ) phase region. It was immediately hot-rolled on a two-roll mill with a 10% reduction per pass. Following each pass, the plate was returned to the furnace and held at 1250 °C for 5 min. A total of 5 rolling passes were performed. After the final pass, the plate was furnace-cooled from 750 °C.

**Figure 1:**
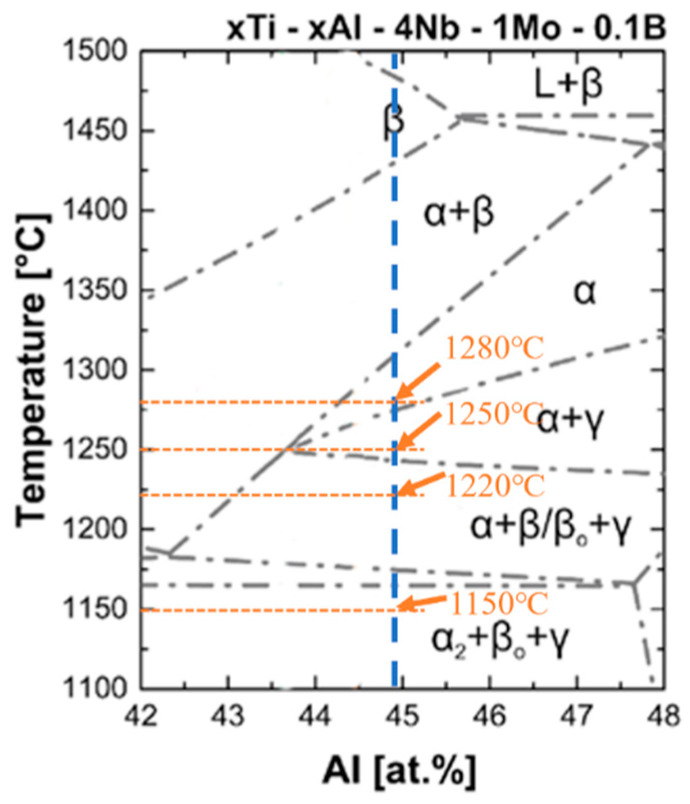
Binary phase diagram of *x*Ti-*x*Al-4Nb-1Mo-0.1B [ at. %].

Cylindrical specimens with dimensions of φ4 mm × 10 mm were cut from the rolled plate using wire cutting. The heat treatment experiments were conducted on a DIL805A thermal dilatometer. To systematically investigate the effect of phase composition and cooling dynamics on microstructure, high-temperature heat treatments were designed based on the TiAl alloy pseudo-binary phase diagram [14], as shown in Figure 1, covering four key phase regions: 1150 °C (α_2_ + βo + γ), 1220 °C (α + β/βo + γ), 1250 °C (α + γ), and 1280 °C (α single-phase region); each temperature was held for 10 min to ensure full phase transformation. For cooling rates, three levels (0.1 °C/s, 0.5 °C/s, and 2.5 °C/s) were selected—referencing industrial cooling process ranges and pre-experiments—to capture the transition of γ lamella nucleation mechanisms and grain boundary morphology. The specific heat treatment parameters are summarized in Table 1.

High-temperature tensile tests were performed using a Gleeble 3500 thermal simulation testing machine (DSI, Poestenkill, NewYork, NY, USA) at a deformation temperature of 800 °C with a strain rate of 1.0 × 10^−4^ s^−1^, at least 3 repeated tests were performed for each condition. Phase analysis of the microstructures was conducted using a Bruker D8 Advance X-ray diffractometer (XRD, Bruker, Karlsruhe, Germany) with a 2θ scanning range of 20° to 90° and a step size of 0.02°. Specimens (heat-treated and tensile-tested) were ground with sandpaper from 200-grit to 2000-grit, followed by mechanical polishing using a SiO_2_ suspension. Microstructural observation was then performed using a QUANTA 450FEG field-emission scanning electron microscope (SEM, FEI Company, Hillsboro, OR, USA). For electron backscatter diffraction (EBSD) sample preparation, the TiAl alloy was electrolytically polished in a solution of 60% methanol + 34% n-butanol + 6% perchloric acid at −25 °C with a voltage of 15–25 V. EBSD analysis was subsequently carried out using a MERLIN COMPACT field-emission SEM(ZEISS, Oberkochen, Germany) equipped with an EBSD detector. In this study, in situ observation of the high-temperature evolution of the material microstructure in rolled TNM-derived alloys was conducted using the High Temperature Laser Scanning Confocal Microscope (HTLSCM, LASERTEC, Yokohama, Japan). HTLSCM samples were cylindrical specimens with a diameter and height of φ3 × 3 mm. Before the experiments, the surfaces of the specimens were ground and polished to ensure flatness during observation.

## 3. Results

### 3.1. Microstructures of the As-Cast and As-Rolled TiAl Alloy

**Figure 2:**
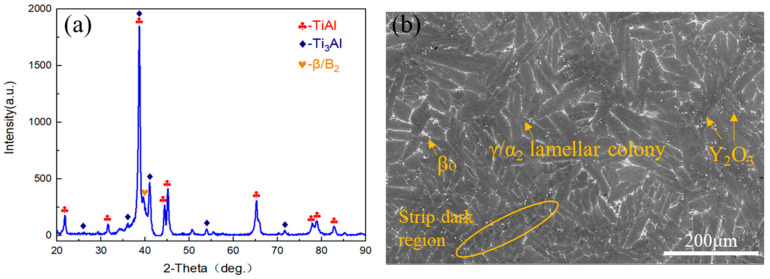
Microstructures of the as-cast TiAl alloy. (**a**) XRD patterns; (**b**) SEM image.

Figure 2a shows the XRD pattern of the as-cast TiAl alloy. It reveals that the TiAl alloy primarily consists of γ-TiAl, α_2_-Ti_3_Al, and β/β_o_ phases. The addition of β-stabilizing elements leads to a higher content of β/β_o_ phases in TiAl alloys compared to conventional γ-TiAl alloys. Figure 2b displays the microstructure of the as-cast TiAl alloy observed in backscattered electron (BSE) mode. The γ-TiAl phase appears dark gray, while the α_2_-Ti_3_Al phase exhibits medium gray, due to differences in atomic number contrast. The low diffusion coefficients of β phase stabilizing elements (Nb, Mo) promote the precipitation of these white β phase particles during solidification. Additionally, the bright white granular features correspond to trace amounts of added Y_2_O_3_. The γ phase, distributed sparingly at lamellar boundaries, predominantly forms γ/α_2_ lamellae with an average colony size of 52 μm. Irregular white β_o_ phase particles are predominantly located at lamellar boundaries. During the β→α phase transformation, the limited solid solubility of β-stabilizing elements (e.g., Nb, Mo) in the α phase induces their segregation to α phase grain boundaries, which stabilizes β phase precipitation at these interfaces. Chemical inhomogeneity results from the solute redistribution during solidification [15]. The enrichment of Al in the liquid phase region induces micro-segregation during solidification, manifested as strip dark regions, as shown in Figure 2b.

**Figure 3:**
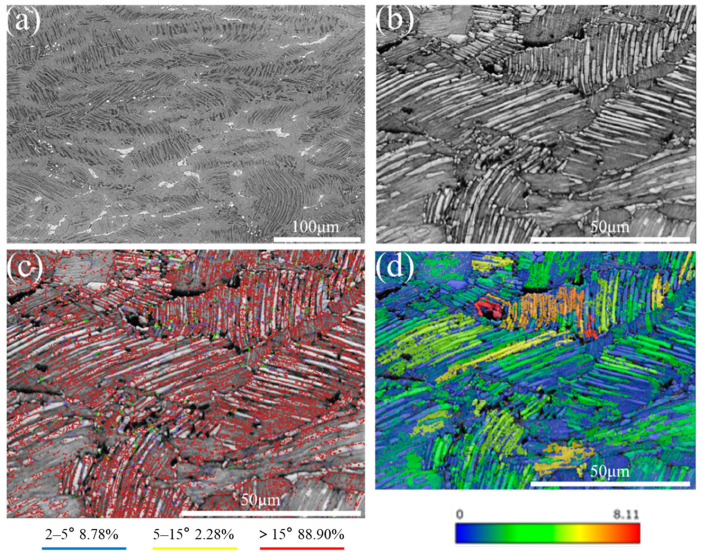
The SEM and EBSD images of TiAl alloy rolled plate. (**a**) SEM; (**b**) contrast map; (**c**) grain boundary distribution map (**d**) GOS map.

Plastic deformation serves as a critical technique for eliminating casting defects and improving the mechanical properties of cast components. Appropriate hot deformation can induce fragmentation of coarse as-cast lamellar colony grains, thereby promoting recrystallization nucleation and the formation of fine and homogeneous grains. This microstructural refinement effectively enhances the strength and toughness of the alloy. Figure 3 shows the microstructural morphology and EBSD maps of the TiAl alloy plate along the RD-ND (rolling direction-normal direction) plane after hot rolling. In Figure 3a, the β_o_ phase becomes elongated along the rolling direction during rolling deformation. This phenomenon arises because the β_o_ phase undergoes a disordering transformation to the high-temperature β phase. The β phase possesses a higher number of active slip systems, enabling it to undergo preferential deformation. This behavior exhibits a “lubricating effect” [16], thereby reducing the deformation resistance during the rolling process. Figure 3b reveals that after hot rolling, the lamellar colonies are elongated along the rolling direction, exhibiting flow-line characteristics, with an average colony size of 20.7 μm which is significantly refined. In both Figure 3a,b, the lamellar colonies display pronounced bending and kinking phenomena, accompanied by a noticeable increase in interlamellar spacing and coarsening of γ laths. Following multi-pass hot rolling, some lamellar colonies undergo fragmentation. As demonstrated in previous research [17], this fragmentation differs from mechanical breakdown. Instead, the discontinuous dynamic recrystallization (DDRX) of γ grains is identified as the dominant mechanism driving the decomposition and fragmentation of lamellar colonies during deformation.

Figure 3c illustrates the grain boundary distribution in the as-rolled microstructure, where misorientation angles are categorized as follows: Low-angle grain boundaries (LAGBs, 2–5°): These indicate the presence of dislocations and subgrain structures. Medium-angle grain boundaries (MAGBs, 5–15°) and high-angle grain boundaries (HAGBs, >15°) are also categorized. A significant proportion of HAGBs (88.9%) is observed within γ grain regions inside and near the lamellar colonies. Discontinuous dynamic recrystallization (DDRX) grains nucleate and grow through grain boundary bulging and the migration of HAGBs, with a high density of HAGBs being a hallmark of DDRX [18]. Notably, curved or kinked regions within lamellar colonies exhibit a dense accumulation of LAGBs. This phenomenon arises because bending/kinking introduces localized stress concentrations within lamellar colonies, thereby promoting dislocation multiplication and subgrain formation. Cheng et al. [19] provided an explanation for the observed kinking and bending phenomena in Ti-43.5Al-8Nb-0.2B alloy during high-temperature compression deformation: Under rolling stress, the elastic bending of lamellar colonies first induces an increase in in-plane shear stress. This triggers the proliferation and dense accumulation of edge dislocations within the plane. Subsequent dislocation climb leads to planar array reorientation to the local curvature. When dislocation arrays intersect, kink boundaries are formed.

Figure 3d presents the Grain Orientation Spread (GOS) map, which quantifies the average angular deviation of crystallographic orientations within individual grains relative to neighboring grains. This map serves as a critical tool for distinguishing between recrystallized and deformed grains. In the GOS map, color gradients represent the intensity of lattice distortion: orange and red hues indicate severe lattice distortions (high dislocation densities), whereas blue tones correspond to low-distortion regions. A direct correlation is observed between the GOS map and the grain boundary distribution shown in Figure 3c. Specifically, grains exhibiting high GOS values (orange/red) predominantly align with LAGBs, suggesting that these regions contain substructures formed during plastic deformation.

The γ grain-rich regions at lamellar colony boundaries exhibit a low stacking-fault energy, which suppresses dislocation cross-slip and climb. Consequently, dislocation pile-ups within these γ grains drive the formation of deformation-induced substructures. These substructures gradually transform into HAGBs during continued deformation, as shown in Figure 3c [20], where high-density HAGBs are observed. This transformation facilitates recrystallized grain nucleation by creating nucleation sites with high stored energy. In the GOS map, this process manifests as clusters of blue γ grains surrounding lamellar colonies, indicating localized recrystallization driven by dislocation redistribution and stress relief.

### 3.2. Microstructures of TiAl Alloy After Different Heat Treatment

The mechanical properties of TiAl alloys are primarily governed by lamellar colony size, interlamellar spacing, and grain boundary morphology. Through optimized heat treatment, key microstructural parameters can be systematically controlled, thereby enhancing the mechanical performance of the alloy.

**Figure 4:**
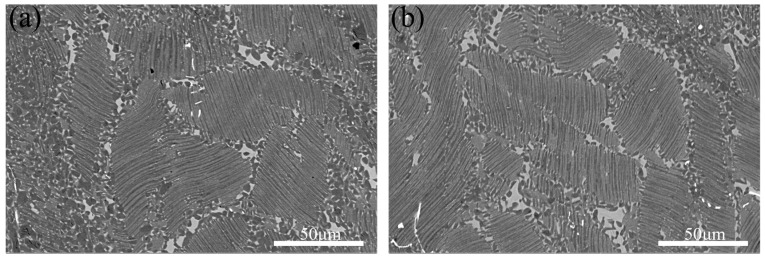
The microstructures of TiAl alloy heat-treated at 1150 °C with different cooling rates. (**a**) 0.1 °C/s; (**b**) 2.5 °C/s.

Figure 4 demonstrates the microstructural evolution of as-rolled TiAl alloy after heat treatment at 1150 °C under varying cooling rates. As shown in Figure 4a,b, the microstructure comprises γ/α_2_ lamellar colonies, blocky β_o_ phase precipitates at lamellar boundaries, and equiaxed γ grains. Figure 4a,b shows the quantitative analysis results using ImageJ software (Image-Pro Plus 6.0) for β_o_ phase content and lamellar colony size. Statistical results reveal that the β_o_ phase content decreases from 6.2% at 0.1 °C/s to 4.1% at 2.5 °C/s, while the average lamellar colony size reduces from 50.6 μm to 40.6 μm. This demonstrates that enhanced cooling rates promote finer lamellar colonies, thereby improving plastic deformation capacity. However, within the (α_2_ + γ + β) three-phase region, cooling rate exhibits negligible influence on interlamellar spacing and lamellar boundary morphology. A comparative analysis of Figure 4a,b reveals that slower cooling rates correlate with increased γ phase fraction at lamellar colony boundaries. This phenomenon arises from the residual strain energy stored within lamellar colony boundaries in the as-deformed microstructure. Under slow cooling conditions, enhanced atomic diffusion facilitates γ phase nucleation from the β_o_ matrix phase.

**Figure 5:**
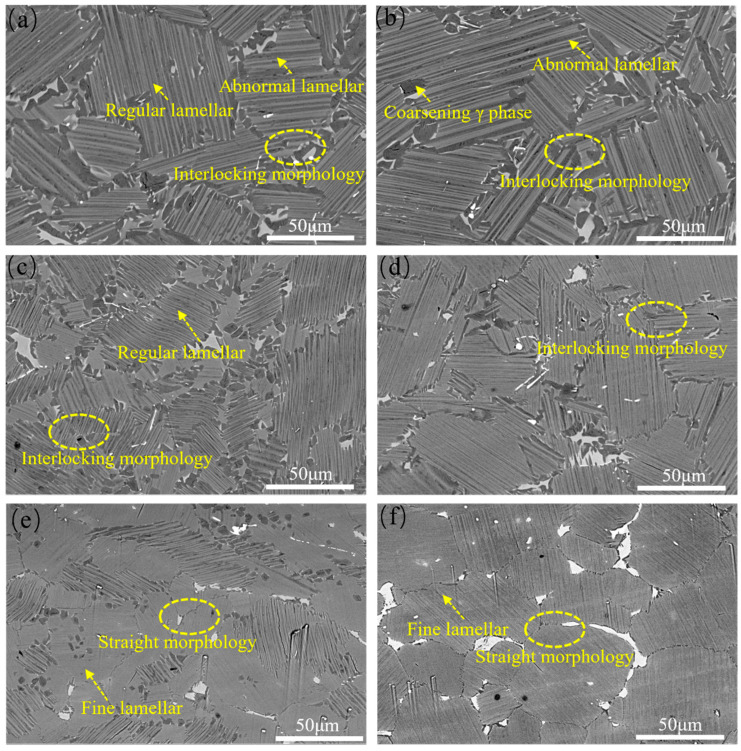
The microstructures of TiAl alloy heat-treated at different conditions. (**a**) 1250 °C, 0.1 °C/s; (**b**) 1280 °C, 0.1 °C/s; (**c**) 1250 °C, 0.5 °C/s; (**d**) 1280 °C, 0.5 °C/s; (**e**) 1250 °C, 2.5 °C/s; (**f**) 1280 °C,2.5 °C/s.

Figure 5 illustrates the microstructural evolution of as-rolled TiAl alloy after heat treatment at 1250 °C and 1280 °C followed by controlled cooling at different rates. At 0.1 °C/s cooling rate, the lamellar colonies exhibit dual-phase microstructure containing both abnormal lamellae (AL) with exceptionally coarse interlamellar spacing and regular lamellae (RL). In contrast, high cooling rates (2.5 °C/s) result in exclusively fine lamellar structures without AL formation. Quantitative analysis using ImageJ software revealed the cooling rate-dependent microstructural evolution: At 1250 °C annealing, interlamellar spacing decreased from 3.2 μm (0.1 °C/s) to 0.7 μm (0.5 °C/s), while the colony size reduced from 57.6 μm (0.1 °C/s) to 36.1 μm (0.5 °C/s). At a cooling rate of 2.5 °C/s, ultrafine γ/α_2_ lamellar structures formed with interlamellar spacing below the SEM resolution limit, and the colony size further decreased to 22.6 μm. Similarly, at 1280 °C, the lamellar spacing decreased from 2.2 μm at a cooling rate of 0.1 °C/s to 0.9 μm at 0.5 °C/s, while the colony size reduced from 54.8 μm (0.1 °C/s) to 36.9 μm (0.5 °C/s).

This phenomenon can be attributed to the increased undercooling at higher cooling rates, which enhances both nucleation and growth rates. Rapid cooling limits the growth time of α and γ phases, leading to significant refinement of the interlamellar spacing. Furthermore, incomplete transformation of the high-temperature α phase to γ during rapid cooling results in a supersaturated α_2_ phase at room temperature, contributing to less distinct α_2_/γ interlamellar boundaries. Cooling rate also influences γ lamellar thickness, which is the primary factor controlling surface undulation amplitude [21]. Consequently, thicker γ lamellae formed at 0.1 °C/s exhibit pronounced surface undulations, whereas the narrower γ lamellae formed at 2.5 °C/s show reduced undulation

Furthermore, cooling rate variations exert a profound influence on the morphological characteristics of lamellar colony boundaries. As demonstrated, higher cooling rates (e.g., 2.5 °C/s) facilitate smoother colony boundaries, whereas slower rates (0.1 °C/s) induce rough, interlocking morphologies. Microstructural observations reveal γ lamellae penetration into adjacent colonies near grain boundaries, accompanied by abnormal γ lamellae growth and even equiaxed γ grain formation, resulting in broader grain boundary regions. The interlocking grain boundary morphology formed under slower cooling rates acts as a mechanical barrier to crack nucleation and propagation at colony boundaries. This enhanced interlocking effectively suppresses grain boundary sliding during steady-state creep, thereby improving intergranular stability and increasing creep resistance by restricting high-temperature grain boundary migration [22].

**Figure 6:**
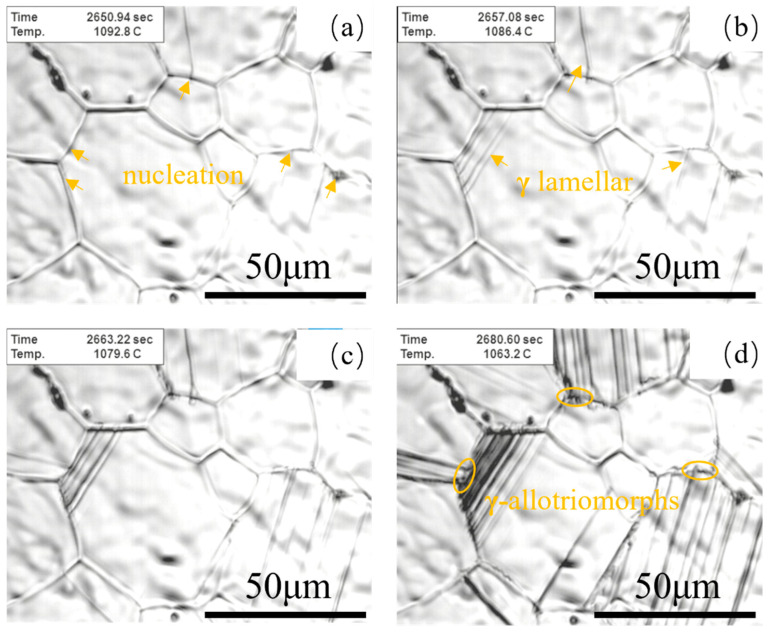
In situ observation of lamellar colony boundary formation. (**a**) 1092.8 °C; (**b**) 1086.4 °C; (**c**) 1079.6 °C; (**d**) 1063.2 °C.

Figure 6 shows the in situ observation images of microstructural evolution in TiAl alloy during slow cooling. Samples underwent a pre-treatment cycle: heating to 1280 °C, followed by 10 min of isothermal holding (to ensure complete phase transformation), then constant-rate cooling at 1 °C/s. The in situ observations during the slow cooling of the TiAl alloy revealed that γ lamellar structures nucleated and grew at grain boundaries, forming γ allotriomorphic structures at their tips. Li et al. [23] established a direct correlation between colony boundary formation and γ allotriomorphic development: following γ phase precipitation at grain boundaries, γ lamellae grow into α grains on one side of the grain boundary, whereas γ allotriomorphic structures form in the α grain regions adjacent to the grain boundary on the opposite side.

**Figure 7:**
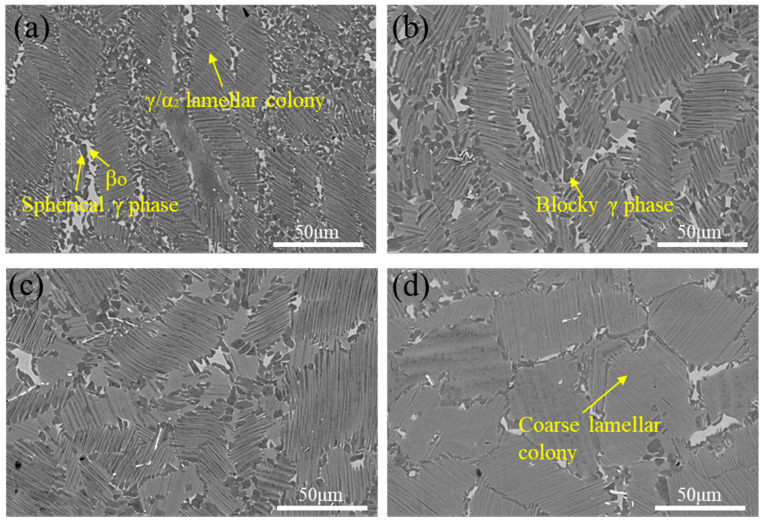
The microstructures of TiAl alloy heat-treated at different temperatures with a cooling rate of 0.5 °C/s. (**a**) 1150 °C; (**b**) 1220 °C; (**c**) 1250 °C; (**d**) 1280 °C.

Figure 7 illustrates the microstructural evolution under different heat treatment temperatures with a constant cooling rate of 0.5 °C/s. Specimens heat-treated within the (α_2_ + β + γ) three-phase region at 1150 °C exhibit microstructures comprising γ/α_2_ lamellar colonies surrounded by fine β_o_ phase and γ phase precipitates, with an average lamellar colony size of 21.2 μm, which is characteristic of a duplex microstructure. Similarly, samples treated at 1220 °C within the same three-phase region display duplex microstructures consisting of γ/α_2_ lamellar colonies and equiaxed β_o_/γ phase networks at colony boundaries, showing slightly larger average lamellar colony sizes (25.5 μm). At 1250 °C (two-phase region), the microstructure comprises γ/α_2_ lamellar colonies with minor β_o_ phase and γ phase precipitates, showing near-lamellar morphology. The average lamellar colony size increases from 21.2 μm (1150 °C) to 33.4 μm (1250 °C). At 1280 °C (α single-phase region), negligible γ phase precipitation near lamellar colonies leads to a fully lamellar microstructure dominated by (α_2_ + γ) colonies, with a residual β_o_ phase at colony boundaries. The lamellar colony size further increases to 41.2 μm, reflecting a coarsened microstructural state.

### 3.3. Mechanical Properties of TiAl Alloy

**Figure 8:**
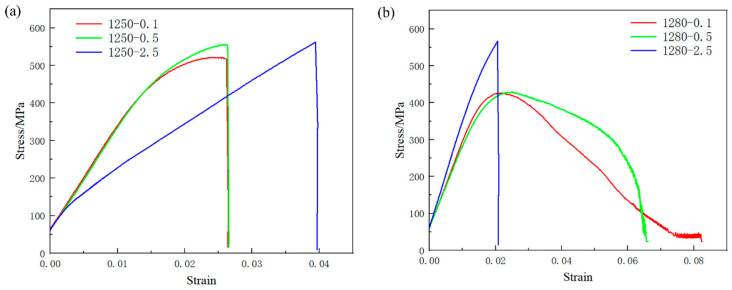
High-temperature tensile stress–strain curves at different heat treatment temperatures. (**a**) 1250 °C; (**b**) 1280 °C.

The stress–strain curves in Figure 8 show that the tested TiAl alloy has ductility below 5%, reflecting its intrinsic brittleness that limits large-scale application. This is because that the dominant γ-TiAl phase has an ordered L1_0_ tetragonal structure, while the α_2_-Ti_3_Al phase has a D0_19_ hexagonal structure. Neither phase possesses a sufficient number of independent slip systems required for extensive plastic deformation. As shown in Figure 8a,b, the ultimate tensile strength (UTS) generally increases with higher cooling rates, though the magnitude of increase depends on the treatment temperature. Notably, at least 3 repeated tests were performed for each condition, and the highest UTS values were achieved at a cooling rate of 2.5 °C/s for both 1250 °C and 1280 °C treatments: at 1250 °C, UTS rises gradually from ~480 MPa (0.1 °C/s) to ~520 MPa (0.5 °C/s) and further to ~550 MPa (2.5 °C/s); at 1280 °C, a more pronounced UTS enhancement is observed, reaching 566 MPa at 2.5 °C/s (from ~410 MPa at 0.1 °C/s). Figure 8a demonstrates relatively low elongation values across all tested conditions, whereas Figure 8b reveals that specimens cooled at 0.1 °C/s and 0.5 °C/s exhibit moderate ductility enhancements. The highest elongation was achieved under the 1280 °C/0.1 °C/s condition.

**Figure 9:**
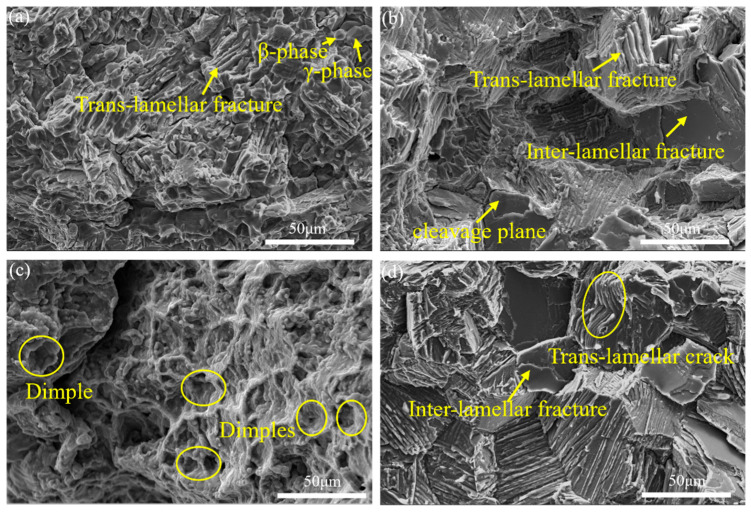
Fracture morphologies under different heat treatment processes. (**a**) 1250–0.1 °C/s; (**b**) 1250–2.5 °C/s; (**c**) 1280–0.1 °C/s; (**d**) 1280–2.5 °C/s.

Figure 9 presents the fracture morphologies of specimens subjected to different heat treatment processes. Figure 9a exhibits small smooth facets characteristic of γ grain cleavage fracture alongside flat trans-granular brittle fracture planes in the β_o_ phase. In contrast, Figure 9b,d display extensive cleavage planes accompanied by prominent interlamellar and trans-lamellar fractures, all indicative of predominantly brittle fracture behavior. Notably, Figure 9c reveals dimpled fracture surfaces with significant plastic deformation features.

**Figure 10:**
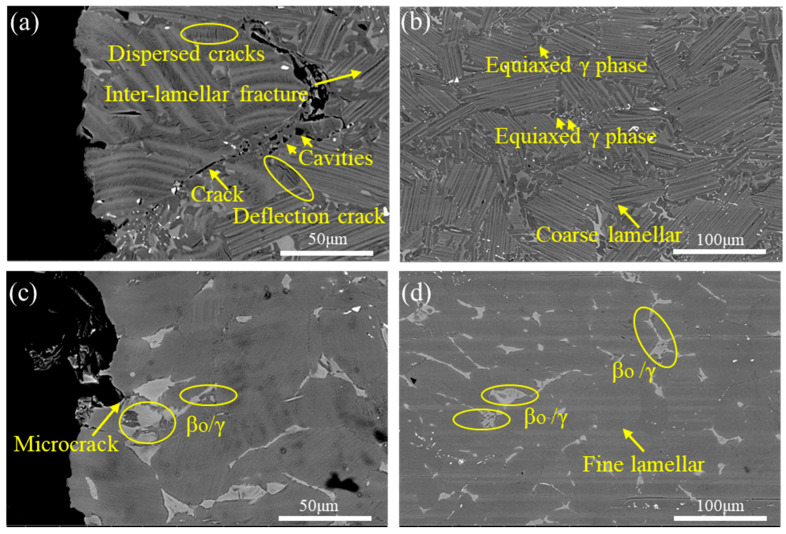
Distal and proximal morphologies of fracture section at different cooling rates. (**a**) 0.1 °C/s, proximal; (**b**) 0.1 °C/s, distal; (**c**) 2.5 °C/s, proximal; (**d**) 2.5 °C/s, distal.

As shown in Figure 10a,b, the microstructures near and far from the fracture surfaces of specimens heat-treated at 1280–0.1 °C/s exhibit a high-volume fraction of equiaxed γ phase with reduced β_o_ phase content. The morphological isotropy of equiaxed γ grains facilitates dislocation glide mechanisms by providing statistically randomized slip pathways. Furthermore, Figure 10a reveals a tortuous crack path characterized by dispersion, deflection, and branching morphologies. This tortuous crack morphology impedes stress concentration by disrupting stress localization, thereby reducing the effective stress intensity factor (SIF). Crack deflection and branching mechanisms increase the effective crack propagation length, enhancing energy absorption during fracture. Consequently, the enhanced damage tolerance significantly delays crack propagation and improves fracture resistance.

As shown in Figure 10c,d, specimens heat-treated at 1280–2.5 °C/s exhibit significantly increased β_o_ phase content near and far from the fracture surfaces, with extensive β_o_ phase accumulations and β_o_/γ mixed microstructures surrounding lamellar colonies. During tensile deformation at 800 °C, incoherent deformation between γ and β_o_ phases initiates crack nucleation. Subsequent crack propagation preferentially follows lamellar colony boundaries, culminating in rapid fracture. Notably, compared to Figure 10a,c, it shows fewer distributed cracks, indicating reduced damage tolerance. This microstructural characteristic serves as the primary contributor to brittle fracture.

## 4. Discussion

### 4.1. Effect of Temperatures on the Microstructure of TiAl Alloys

At a heat treatment temperature of 1150 °C (within the α_2_ + β_o_ + γ three-phase region), subsequent cooling does not induce phase transformations. The presence of abundant β_0_ and γ phases surrounding the lamellar colonies restricts their coarsening during solidification. Similarly, heat treatment at 1220 °C (within the α_2_ + β_o_ + γ three-phase region) also suppresses lamellar colony growth due to the persistent stabilization of β_o_ and γ phases at lamellar interfaces. However, compared to the 1150 °C treatment, elevated heat treatment temperatures result in reduced β_o_ phase content and increased γ phase precipitation at lamellar colony boundaries. This phenomenon arises from accelerated atomic diffusion at higher temperatures, which promotes continuous γ phase nucleation and growth from the β_o_ phase. As adjacent γ phases coarsen and merge during cooling, enlarged γ grains are formed through Ostwald ripening mechanisms.

When the heat treatment temperature is elevated to 1250 °C, the phase diagram indicates the material resides in a two-phase region slightly below the T_α_ transition temperature. Proximity to the T_α_ transition temperature promotes an increased γ→α transformation with rising temperature, resulting in a significant reduction in equiaxed γ grain content compared to the three-phase region. The enhanced α phase fraction facilitates substantial growth of lamellar colonies during subsequent cooling. Concurrently, diminished constraints from reduced equiaxed γ grains allow notable expansion in average lamellar colony size. When the heat treatment temperature is further increased to 1280 °C, the system enters the α single-phase region. Under these conditions, β-stabilizing elements (Nb, Mo) undergo sufficient diffusion, leading to enhanced solute redistribution and gradual homogenization. Consequently, the β-phase content decreases to 0.72%, further diminishing the constraints on lamellar colony growth. The accelerated grain boundary migration rate triggers a dramatic coarsening of microstructural features, resulting in an average lamellar colony size of 41.2 μm. Notably, the interlamellar spacing exhibits no significant variation trend during this thermal treatment, demonstrating that heating temperature is not a primary factor governing interlamellar spacing evolution.

### 4.2. Effect of Cooling Rates on the Microstructure of TiAl Alloys

#### 4.2.1. Effect of Cooling Rates on the Interlamellar Spacing

As evidenced by Figure 4 and Figure 5, the interlamellar spacing exhibits strong sensitivity to cooling rate, with increasing cooling rate leading to pronounced refinement of the lamellar structure. Researchers have established the following empirical relationship between interlamellar spacing (*λ*) and cooling rate ($\dot{v}$) [24]:

(1) $$\lambda =K{\dot{v}}^{a}$$

where α represents the cooling rate exponent (ranging from −0.42 to −0.45 based on regression analysis of experimental data), *K* denotes a material constant, *λ* is the interlamellar spacing, and $\dot{v}$ indicates cooling rate (°C/min). This formulation demonstrates an inverse correlation between cooling rate and lamellar spacing, where accelerated cooling promotes progressive refinement of the lamellar structure, a trend that aligns well with the experimental observations in the present study. Lapin’s research [24] demonstrates that γ lamellae formation within α phase TiAl alloys is governed by long-range diffusion processes. The stepwise lamellae nucleation mechanism involves dislocation emission from the α phase matrix and prism-to-basal plane cross-slip events, leading to stair-step stacking fault configurations. Increased cooling rates suppress dislocation cross-slip capabilities at prism/basal interfaces, thereby restricting atomic mass transfer across ledge-kink defects and inhibiting growth of γ lamellae. Under a cooling rate of 2.5 °C/s, TiAl alloys develop the finest lamellar structure with maximized volume fraction of non-equilibrium α_2_ phase. In contrast, at 0.1 °C/s cooling rate, dislocations undergo repeated basal plane pinning, enabling extensive nucleation of adjacent γ grains. The enhanced diffusion kinetics under slow cooling facilitates lateral growth of γ grains into coarse laths, resulting in a near-equilibrium phase fraction.

The elevated defect density at grain boundaries facilitates preferential γ phase nucleation along α grain boundaries, as evidenced in Figure 6a. Following nucleation, rapid longitudinal growth of γ lamellae occurs along established lamellar interfaces (Figure 6b). Subsequent cooling promotes γ phase precipitation adjacent to existing lamellae, forming twin-related crystallographic configurations. Crucially, this twinned growth mode requires lower activation energy compared to homogeneous α matrix nucleation, attributable to coherent twin boundaries providing minimal interfacial energy. It was revealed in the experimental study that the elevated cooling rate substantially augmented both the population density of γ lamellae precipitates and their growth kinetics along the longitudinal direction. This microstructural evolution induced localized stress accumulation within adjacent matrix domains, thereby facilitating the direct precipitation of fresh γ-lamellae near pre-existing ones. Upon reaching a critical cooling rate of 120 °C/min, the adjacent precipitation mechanism became predominant [23]. Notably, under such rapid cooling conditions, the initial precipitation stage exhibited dual characteristics: (i) conventional nucleation along α phase boundaries, and (ii) a substantial volume fraction of intragranular γ lamellae precipitates.

**Figure 11:**
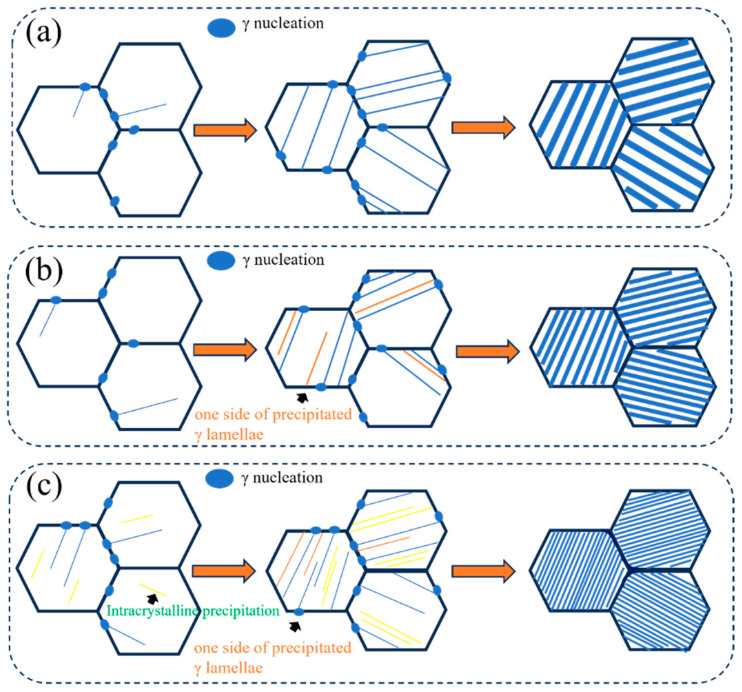
The schematic diagram of γ nucleation and growth at different cooling rates. (**a**) 0.1 °C/s; (**b**) 0.5 °C/s; (**c**) 2.5 °C/s.

Based on the above analysis, the schematic illustration of γ precipitation and growth is shown in Figure 11. The precipitation mechanisms of γ lamellae under different cooling rates can be summarized as follows: At an extremely low cooling rate of 0.1 °C/s, γ precipitates nucleate almost exclusively at α grain boundaries, followed by growth (Figure 11a). When the cooling rate increases to 0.5 °C/s, γ lamellae not only nucleate and grow at α grain boundaries but also form and grow adjacent to pre-existing γ lamellae (Figure 11b). When the cooling rate increases to 2.5 °C/s, the nucleation and growth of γ phase occur not only at α grain boundaries and adjacent to pre-existing γ lamellae, but also within α grains due to the higher undercooling caused by the elevated cooling rate. The substantial increase in nucleation sites restricts the sufficient thickening growth of γ lamellae along the thickness direction, consequently resulting in finer final interlamellar spacing.

#### 4.2.2. Effect of Cooling Rates on the Grain Boundary Morphology

**Figure 12:**
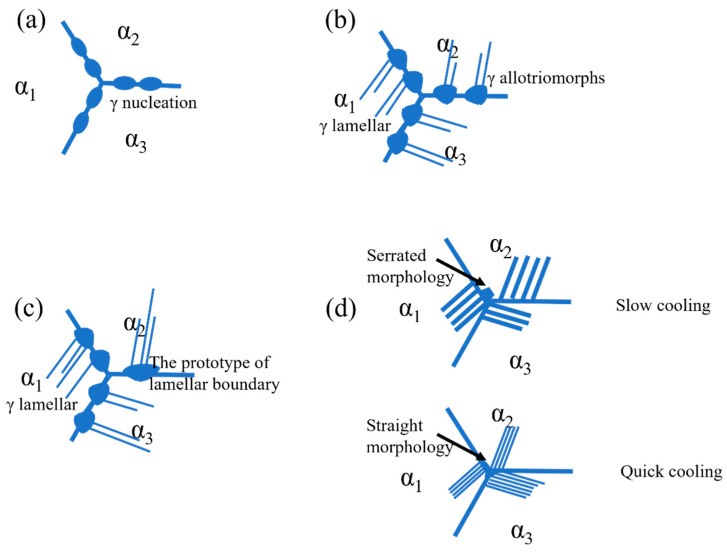
Schematic diagram of lamellar boundary morphology formation. (**a**) γ nucleation; (**b**) The formation of γ allotriomorphs; (**c**) The prototype of lamellar boundary; (**d**) Grain boundary morphology at different cooling rates.

Zghal et al. [25] found that the formation of γ allotriomorphic structure exhibits a strong temperature dependence. The slower the cooling rate, the more extensive the growth range of γ allotriomorphic structure. Moreover, the formation of lamellar colony boundaries is closely associated with the growth of allotriomorphic structure. Figure 12 illustrates the morphological evolution of lamellar boundaries. Based on in situ observation results shown in Figure 6, γ-phase nucleation and growth most readily occur at grain boundaries. The α grains at grain boundaries are designated as α_1_, α_2_, and α_3_, respectively, as illustrated in Figure 12a. In Figure 12b, the nucleating γ phase exhibits sluggish and discontinuous growth when penetrating α_2_ grains, whereas it displays planar lamellar morphology with significantly accelerated growth rates within α_1_ grains. At this stage, the γ phase in α_1_ grains establishes a Blackburn orientation relationship with the α_1_ matrix. The regions where γ phase cease to propagate within the grains causes the appearance of the γ allotriomorphs. The coalescence and growth of γ allotriomorphs near grain boundaries initiate the formation of incipient lamellar colony boundaries, as shown in Figure 12c. Under extremely slow cooling conditions, these incipient boundaries develop fully into morphologically distinct features characterized by roughened interfaces. In certain cases, petal-like contours emerge, resulting in serrated boundary morphologies. As cooling rates increase, partial suppression of incipient boundary growth occurs alongside reduced grain boundary widths. Some grain boundaries exhibit straightened morphologies, as observed in Figure 12d.

### 4.3. Effect of Microstructural Parameters on the Mechanical Properties of TiAl Alloys

Increased cooling rates result in reduced interlamellar spacing and modest decreases in colony size. According to the Hall-Petch relationship, finer interlamellar spacing enhances strength through increased resistance to dislocation motion.

(2) $$\sigma_{s}=\sigma_{0}+k_{d}D^{-0.5}+k_{\lambda }\lambda^{-0.5}$$

where the *σ_s_* is yield strength, *σ_0_* is material constant, *D* is colony size, *λ* is the interlamellar spacing, *k_d_* and *k_λ_* represent the efficient governing colony size and interlamellar spacing effects, respectively.

This strengthening mechanism arises from the abundance of semi-coherent interfaces in fine-grained TiAl alloys, which act as dislocation barriers. Dislocation pile-up at these semi-coherent interfaces necessitates higher applied stresses to sustain plastic deformation during tensile tests, thereby improving ultimate tensile strength. The experimental data confirms this trend: a 140 MPa reduction in UTS was observed at 1280 °C heat treatment between 0.1 °C/s (426 MPa) and 2.5 °C/s (566 MPa) cooling rates. The reduction in lamellar colony size enhances the capacity for plastic deformation to a certain extent. This phenomenon is attributed to the increased grain boundary density resulting from lamellar colony refinement, which facilitates coordinated deformation in fine-grained microstructures and thereby improves plasticity.

In high-temperature tensile mechanical properties, elongation is significantly influenced by β_o_ phase content rather than cooling rate or lamellar spacing. Reduced β_o_ phase content and uniformly distributed equiaxed γ phases around lamellae effectively enhance elongation. This behavior arises because γ phase acts as a soft phase during high-temperature tensile testing at 800 °C, whereas β_o_ phase serves as a hard phase. The incoherent deformation between γ and β_o_ phases promotes microcrack initiation, while the hard β_o_ phase itself frequently acts as a crack nucleation site. Consequently, minimizing β_o_ phase content is critical for improving ductility.

Equiaxed γ grains surrounding lamellar colonies exhibit isotropic characteristics, which enhance multiple deformation mechanisms such as dislocation glide. These mechanisms provide additional slip pathways; however, when dislocation density exceeds a critical threshold, dislocation pile-up occurs. It is noteworthy that the face-centered cubic (FCC) structure of γ-TiAl phase effectively mitigates dislocation pile-up density caused by increased dislocation density, thereby enhancing its plastic deformation capacity. Specifically, higher equiaxed γ grain density and more uniform distribution correlate directly with improved elongation. Xue et al. [26] further demonstrated the positive correlation between equiaxed γ phase and elongation improvement. During tensile deformation, equiaxed γ grains exhibited abundant slip lines with continuous slip band propagation, which play a critical role in continuous stress relief and stress concentration mitigation. Yue et al. [27] revealed that nano-Y_2_O_3_ addition increases the “soft” γ phase content within lamellar colonies and at colony boundaries. Concurrently, the emergence of dislocation glide and twinning activity in equiaxed γ phases during tensile tests indicates significant plastic deformation in these regions. This enhanced ductility effectively alleviates stress concentration and promotes homogeneous plastic flow, thereby improving the overall plasticity of TiAl alloys.

From the perspective of grain boundary morphology, straighter grain boundaries imply lower resistance to crack propagation during post-nucleation stages, leading to rapid fracture and inferior plasticity. In contrast, when grain boundaries exhibit cross-interlocked structures, cracks undergo deflection, bridging, and other energy-dissipating mechanisms during propagation, thereby delaying fracture initiation and macroscopically enhancing plasticity. Therefore, critical microstructural parameters should be considered: (1) Obtaining appropriately refined lamellar colonies and interlamellar spacing improves strength; (2) Cross-interlocked grain boundary morphologies and uniformly dispersed equiaxed γ grains at the boundaries enhance plasticity; (3) The presence of brittle β_o_ phase should be minimized.

The preceding analysis clarifies TiAl alloy’s “processing-microstructure-performance” relationship, but deeper interpretation, limitations, and future directions merit attention. A key finding is the decoupling of temperature and interlamellar spacing: temperature governs colony size (21.2–41.2 μm) via phase thermodynamics, yet interlamellar spacing depends on cooling kinetics—guiding the fact that refining spacing needs cooling rate optimization. Equiaxed γ grains enhance plasticity but excessive growth weakens colony coarsening resistance, requiring precise fraction control.

This study has limitations: narrow cooling range (0.1–2.5 °C/s, no extreme quenching), lack of long-term performance tests (e.g., creep for aero-engines), and incomplete fast-cooling in situ observations. Future work should explore ultra-fast cooling, test long-term creep properties, and use updated technologies to clarify intragranular nucleation kinetics, promoting industrial application.

## 5. Conclusions

(1) During the rolling deformation process, the high-temperature disordered β phase possesses abundant slip systems and undergoes preferential deformation, acting as a “lubricating effect” to reduce flow stress during rolling. Lamellar colonies elongate along the deformation direction and may even fragment or decompose. In TiAl alloys, discontinuous dynamic recrystallization of γ grains is the dominant mechanism driving lamellar colony fragmentation during deformation.
(2) When the heat treatment temperature is within the (α_2_ + β_0_ + γ) three-phase region, a higher volume fraction of β_0_ and γ phases exists around the lamellar colonies, restricting their coarsening. As the heat treatment temperature increases, γ phase continuously precipitates from the β_o_ phase, leading to a gradual reduction in β_o_ phase content. When the temperature rises to Tₐ, γ phase progressively transforms into α phase, further decreasing the β_0_ and γ phases that constrain colony growth while increasing the volumetric fraction of lamellar colonies. At 1280 °C, a nearly fully lamellar microstructure is obtained, with an average colony size approaching 41.2 μm. However, elevated temperatures have minimal impact on interlamellar spacing.
(3) Cooling rate is a critical factor influencing lamellar colonies; higher cooling rates result in finer interlamellar spacing. Additionally, the precipitation behavior of γ lamellae is closely tied to the cooling rate. At extremely low cooling rates (0.1 °C/s), γ lamellae nucleate and grow exclusively at α grain boundaries. As the cooling rate increases, γ phase nucleates not only at α grain boundaries but also on the sides of pre-existing γ lamellae. At faster cooling rates, such as 2.5 °C/s, homogeneous nucleation of γ phase occurs within the lamellae. The morphology of lamellar colony boundaries is also strongly dependent on the cooling rate. At slow cooling rates, γ allotriomorphs merge and grow near grain boundaries, forming a zigzag interlocking morphology. When the cooling rate increases, γ allotriomorph growth is suppressed, and the colony boundaries transition from a zigzag interlocking to a smooth, straight morphology.
(4) Fine lamellar colony sizes and interlamellar spacing effectively enhance tensile strength, where the relationship between tensile strength and interlamellar spacing is most critical: the smaller the interlamellar spacing, the higher the tensile strength. Brittle β_o_ and γ phases are prone to incoherent deformation, leading to rapid crack nucleation and propagation; thus, brittle β_o_ phases should be eliminated. Zigzag interlocking lamellar boundaries and uniformly distributed equiaxed γ grains inhibit crack propagation and improve plasticity.

## Figures and Tables

**Table 1:** High-temperature heat treatment parameters.

Samples	Temperatures	Holding Times	Cooling Rates
1	1150 °C	10 min	0.1 °C/s
2	1150 °C	10 min	2.5 °C/s
3	1220 °C	10 min	0.5 °C/s
4	1250 °C	10 min	0.1 °C/s
5	1250 °C	10 min	0.5 °C/s
6	1250 °C	10 min	2.5 °C/s
7	1280 °C	10 min	0.1 °C/s
8	1280 °C	10 min	0.5 °C/s
9	1280 °C	10 min	2.5 °C/s

## Data Availability

The original contributions presented in this study are included in the article. Further inquiries can be directed to the corresponding author.
